# Alterations in Kynurenine and NAD^+^ Salvage Pathways during the Successful Treatment of Inflammatory Bowel Disease Suggest HCAR3 and NNMT as Potential Drug Targets

**DOI:** 10.3390/ijms222413497

**Published:** 2021-12-16

**Authors:** Artur Wnorowski, Sylwia Wnorowska, Jacek Kurzepa, Jolanta Parada-Turska

**Affiliations:** 1 Department of Biopharmacy, Medical University of Lublin, 20-093 Lublin, Poland; 2 Department of Medical Chemistry, Medical University of Lublin, 20-093 Lublin, Poland; 45820@student.umlub.pl (S.W.); jacek.kurzepa@umlub.pl (J.K.); 3 Department of Rheumatology and Connective Tissue Diseases, Medical University of Lublin, 20-090 Lublin, Poland; jolanta.parada-turska@umlub.pl

**Keywords:** metabolic network reconstruction, G protein-coupled receptor 109B, immune-mediated inflammatory disease, gut inflammation, anti-cytokine monoclonal antibodies, nicotinamide phosphoribosyltransferase

## Abstract

A meta-analysis of publicly available transcriptomic datasets was performed to identify metabolic pathways profoundly implicated in the progression and treatment of inflammatory bowel disease (IBD). The analysis revealed that genes involved in tryptophan (Trp) metabolism are upregulated in Crohn’s disease (CD) and ulcerative colitis (UC) and return to baseline after successful treatment with infliximab. Microarray and mRNAseq profiles from multiple experiments confirmed that enzymes responsible for Trp degradation via the kynurenine pathway (IDO1, KYNU, IL4I1, KMO, and TDO2), receptor of Trp metabolites (HCAR3), and enzymes catalyzing NAD^+^ turnover (NAMPT, NNMT, PARP9, CD38) were synchronously coregulated in IBD, but not in intestinal malignancies. The modeling of Trp metabolite fluxes in IBD indicated that changes in gene expression shifted intestinal Trp metabolism from the synthesis of 5-hydroxytryptamine (5HT, serotonin) towards the kynurenine pathway. Based on pathway modeling, this manifested in a decline in mucosal Trp and elevated kynurenine (Kyn) levels, and fueled the production of downstream metabolites, including quinolinate, a substrate for de novo NAD^+^ synthesis. Interestingly, IBD-dependent alterations in Trp metabolites were normalized in infliximab responders, but not in non-responders. Transcriptomic reconstruction of the NAD^+^ pathway revealed an increased salvage biosynthesis and utilization of NAD^+^ in IBD, which normalized in patients successfully treated with infliximab. Treatment-related changes in NAD^+^ levels correlated with shifts in nicotinamide N-methyltransferase (NNMT) expression. This enzyme helps to maintain a high level of NAD^+^-dependent proinflammatory signaling by removing excess inhibitory nicotinamide (Nam) from the system. Our analysis highlights the prevalent deregulation of kynurenine and NAD^+^ biosynthetic pathways in IBD and gives new impetus for conducting an in-depth examination of uncovered phenomena in clinical studies.

## 1. Introduction

Inflammatory bowel diseases (IBD) such as Crohn’s disease (CD) and ulcerative colitis (UC) are characterized by disturbed gut homeostasis, leading to chronic inflammation. The prevalence of IBD continuously rises in industrialized societies, especially across individuals with genetic susceptibility who are exposed to certain dietary and environmental factors [1]. However, the exact etiology and pathophysiology of IBD remains unknown.

For many years, the treatment options for IBD were glucocorticoids, immunomodulators (e.g., methotrexate), 5-aminosalicylic acid, cyclosporine, and antibiotics. Later, our understanding of immune responses improved greatly, bringing us new therapeutic options [2]. The introduction of anticytokine therapies revolutionized the management of chronic inflammatory diseases, including IBD. Current CD and UC treatment recommendations, formulated by medical societies and other representative bodies, include the use of anticytokines [3,4]. Infliximab, an anti-TNFα agent, was approved for IBD in 1998 and is still commonly used [5,6]. Subsequently, other human monoclonal antibodies interacting with this cytokine were introduced, i.e., adalimumab, certolizumab, and golimumab [7]. The anti-TNFα drugs induce and maintain remission in moderate to severely active UC and pro-inflammatory CD. Anti-TNFα therapy has been proven to be highly effective in most patients, however, it has disadvantages, such as high costs, serious side effects, and the inconvenience of frequent injections [8]. Therefore, the identification and implementation of small-molecule drugs acting on processes initiated and regulated by cytokines is awaited.

To better understand the pathological cascades affected in IBD, we performed gene expression data mining followed by metabolic pathway reconstruction. The goal was to identify and model the metabolic pathways that are affected during both disease progression and post-treatment recovery. The conducted analyses helped us to identify tryptophan (Trp) metabolism as a disease- and treatment-related pathway in IBD. Furthermore, hydroxycarboxylic acid receptor 3 (HCAR3) and nicotinamide N-methyltransferase (NNMT) were recognized as potential drug targets for reverting IBD-related changes in mucosal levels of Trp, kynurenine (Kyn), nicotinamide adenine dinucleotide (NAD^+^), and related metabolites.

## 2. Results

### 2.1. IDO1, KYNU and HCAR3 Are Upregulated in IBD and Suppressed upon Effective Infliximab Treatment

**Figure 1:**
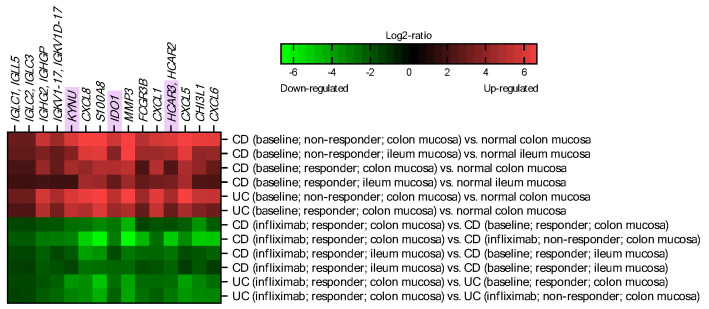
Genes differentially regulated during the course and treatment of inflammatory bowel disease (IBD). Genes identified to undergo marked upregulation in IBD and to be markedly downregulated in response to successful infliximab treatment (|log_2_-ratio| ≥ 1.6). Genes from kynurenine pathway are highlighted in purple. IBD, inflammatory bowel disease; CD, Crohn’s disease; UC, ulcerative colitis.

Publicly available microarray-derived data on gene expression in human intestinal mucosa were exploited for this analysis. Datasets were searched for genes that were markedly deregulated in CD and UC patients and that were returning to baseline only in response to the successful treatment with infliximab. In other words, we searched for genes that were either: (a) down-regulated in IBD patients (irrespective of their responsiveness to infliximab) compared to normal controls and up-regulated in IBD patients successfully treated with infliximab, compared to baseline and non-responders, or (b) up-regulated in IBD patients (irrespective of their responsiveness to infliximab) compared to normal controls and down-regulated in IBD patients successfully treated with infliximab, compared to baseline and non-responders. No significantly affected genes matching the first set of criteria were identified. A later query revealed 15 probes up-regulated in IBD that were suppressed in infliximab responders (Figure 1). The identified probes correspond to genes related to immunoglobulins (*IGLC1*, *IGLL5*, *IGLC2*, *IGLC3*, *IGHG2*, *IGHGP*, *IGKV1-17*, *IGKV1d-17*, *FCGR3B*), cytokines and inflammatory mediators (*CXCL8*, *S100A8*, *CXCL1*, *CXCL5*, *CXCL6*, *CHI3L1*), and tissue remodeling (*MMP3*). In addition, three genes related to Trp metabolism along the kynurenine pathway were identified: *IDO1* (indoleamine 2,3-dioxygenase 1), *KYNU* (kynureninase), and *HCAR3*. *IDO1* and *KYNU* encode enzymes catalyzing Trp degradation and formation of its bioactive metabolites, commonly referred to as kynurenines [9]. *HCAR3* codes for a receptor for kynurenic acid (Kyna), one of the Trp metabolites [10]. Based on this finding, one may conclude that Trp metabolism is particularly perturbed in IBD.

### 2.2. The Kynurenine and NAD^+^ Pathways Show a Consistent Imbalance in IBD

**Figure 2:**
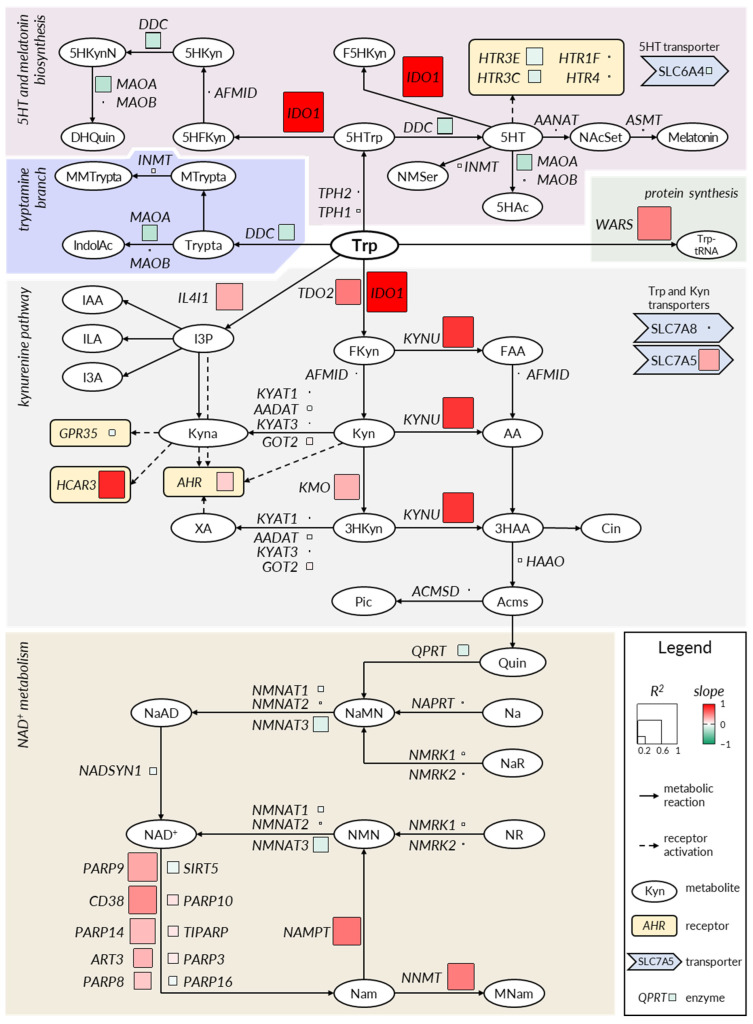
Pathways of tryptophan metabolism. Linear regression of log_2_ fold-change of *IDO1* gene expression ratios vs. other genes related to tryptophan metabolism along kynurenine pathway was calculated and marked with squares of varying size (denoting R^2^ values) and color (indicating slope). Metabolites: AA, anthranilic acid; Trp, tryptophan; Kyn, kynurenine; FKyn, formyl-kynurenine; FAA, formyl-anthranilic acid; 3HKyn, 3-hydroxy-kynurenine; Kyna, kynurenic acid; XA, xanthurenic acid; Acms, 2-amino-3-carboxymuconate semialdehyde; Pic, picolinic acid; Cin, cinnabarinic acid; 3HAA, 3-hydroxyanthranilic acid; 5HTrp, 5-hydroxy-tryptophan; Trypta, tryptamine; MTrypta, methyl-tryptamine; MMTrypta, dimethyl-tryptamine; IndolAc, indole-3-acetaldoxime; 5HFKyn, 5-hydroxy-N-formylkynurenine; 5HKyn, 5-hydroxy-kynurenine; 5HKynN, 5-hydroxykynuramine; DHQuin, 4,6-dihydroxy-quinoline; 5HAc, 5-hydroxy-indoleacetaldehyde; F5HKyn, formyl-5-hydroxykynurenamine; NMSer, N-methyl-serotonin; NAcSet, N-acetyl-serotonin; 5HT, 5-hydroxytryptamine (serotonin); I3P, indole-3-pyruvic acid; IAA, indole-3-acetic acid; I3A, indole-3-aldehyde; ILA, indole-3-lactic acid; Quin, quinolinic acid; Nam, nicotinamide; NAD, nicotinamide adenine dinucleotide; NMN, Nam mononucleotide; NR, Nam riboside; MNam, methyl-Nam; Na, nicotinic acid; NaR, Na riboside; NaAD, Na adenine dinucleotide; NaMN, nicotinic acid mononucleotide. Enzyme-coding genes: *AFMID*, arylformamidase; *KYNU*, kynureninase; *ACMSD*, 2-amino-3-carboxymuconate semialdehyde-decarboxylase; *QPRT*, quinolinic acid phosphoribosyltransferase; *IL4I1*, interleukin 4 induced 1; *AANAT*, arylalkylamine N-acetyltransferase; *ASMT*, acetylserotonin N-methyltransferase; *MAOA/B*, monoamine oxidase A/B; *INMT*, indolethylamine N-methyltransferase; *WARS*, tryptophanyl-aminoacyl-tRNA synthetase; *IDO1*; indoleamine 2,3-dioxygenase 1; *TOD2*, tryptophan 2,3-dioxygenase; *DDC*, aromatic l-amino acid decarboxylase; *TPH1/2*, tryptophan hydroxylase 1/2; *KYAT1*, kynurenine aminotransferase 1; *AADAT*, aminoadipate aminotransferase; *KYAT3*, kynurenine aminotransferase 3; *GOT2*, glutamic-oxaloacetic transaminase 2; *KMO*, kynurenine 3-monooxygenase; *HAAO*, 3-hydroxyanthranilate 3,4-dioxygenase; *NAPRT*, nicotinate phosphoribosyltransferase; *NMNAT1/2/3*, nicotinamide nucleotide adenylyltransferase 1/2/3; *NMRK1/2*, nicotinamide riboside kinase 1/2; *NAMPT*, nicotinamide phosphoribosyltransferase; *NNMT*, nicotinamide N-methyltransferase; NADSYN1, glutamine-dependent NAD^+^ synthetase; *PARP*, poly(ADP-ribose) polymerase; *CD38*, cyclic ADP-ribose hydrolase; *ART3*, ecto-ADP-ribosyltransferase 3; *SIRT5*, sirtuin 5; *TIPARP*, TCDD-inducible poly(ADP-ribose) polymerase. Transporter-coding genes: *SLC7A5*, large neutral amino acids transporter small subunit 1; *SLC7A8*, large neutral amino acids transporter small subunit 2; *SLC6A4*, serotonin transporter (SERT or 5-HTT). Receptor-coding genes: *GPR35*, G protein-coupled receptor 35; *AHR*, aryl hydrocarbon receptor; *HCAR3*, hydroxycarboxylic acid receptor 3 (GPR109B); *HTR*, 5-hydroxytryptamine receptor. Diagram was prepared based on the following studies: [12,13,14].

In mammals, Trp metabolism occurs via three key branches: (I) the 5HT (serotonin) pathway, (II) protein biosynthesis, and (III) the kynurenine pathway; moreover, the kynurenine pathway supplies fuel for de novo production of NAD^+^ (Figure 2). Other minor branches included Trp decarboxylation to tryptamine, IL4I1-mediated indole formation, and conversion of 5HT to melatonin. IDO1 is a key enzyme of Trp metabolism—it is the first and rate-limiting enzyme guiding Trp degradation towards kynurenines, consequently reducing Trp’s availability for other pathways [11]. As *IDO1* expression is substantially regulated in the intestinal mucosa during the course and treatment of IBD (Figure 1), we conducted a correlation analysis involving *IDO1* and all other genes related to Trp metabolism (Figure 2, Supplementary Figure S1). Microarray (*n* = 204) and RNAseq (*n* = 153) studies were analyzed. The aim was to establish which genes are co-regulated with *IDO1* in the context of IBD. For each gene of interest within every study, the log_2_-ratio was calculated as the difference between the mean log_2_ expression for experimental samples and the mean log_2_ expression for corresponding control samples from the same experiment. Subsequently, log_2_-ratios for *IDO1* were plotted against log_2_-ratios for every other gene contributing to the kynurenine pathway (Supplementary Figure S1). Slope and R^2^ values from linear regression were marked on the Trp metabolism diagram (Figure 2). In IBD, the changes in the expression of *IDO1* correlated positively with the changes in the expression of *KYNU* (slope = 0.79; R^2^ = 0.76), *HCAR3* (slope = 0.84; R^2^ = 0.69), IL4I1 (slope = 0.32; R^2^ = 0.67), *KMO* (slope = 0.30; R^2^ = 0.63), and *TDO2* (slope = 0.52; R^2^ = 0.62). When the analysis was performed for intestinal neoplasm samples, there was no correlation in the change in IDO1 expression vs. *KYNU* (slope = 0.10; R^2^ = 0.04), *HCAR3* (slope = 0.37; R^2^ = 0.09), IL4I1 (slope = 0.35; R^2^ = 0.24), *KMO* (slope = 0.24; R^2^ = 0.11), nor *TDO2* (slope = 0.70; R^2^ = 0.21) (Supplementary Figure S1). This suggests selective and synchronous deregulation of the kynurenine pathway in IBD.

A similar pattern of gene expression deregulation was observed in the context of some key enzymes of the NAD^+^ salvage pathway (Figure 2, Supplementary Figure S1). In IBD, changes in *IDO1* expression significantly correlated with the modulation of *NAMPT* (slope = 0.54; R^2^ = 0.69) and *NNMT* (slope = 0.51; R^2^ = 0.66) as well as *PARP9* (slope = 0.34; R^2^ = 0.72), *CD38* (slope = 0.44; R^2^ = 0.69), and several other NAD-consuming enzymes. The positive correlations were not recapitulated in the context of intestinal neoplasms (Supplementary Figure S1). The correlation analysis indicates that *IDO1* is co-regulated with the key enzymes involved in the biosynthesis and turnover of NAD^+^. This orchestrated co-regulation occurs in the intestine in the context of IBD, but not in the context of malignancies.

No significant correlations were observed between the changes in *IDO1* expression and changes in the expression of genes involved in 5HT, melatonin, and tryptamine pathways.

### 2.3. Deregulation in Gene Expression Shifts the Tryptophan Metabolite Landscape from Serotonin Production towards Kynurenine Pathway and NAD^+^ Synthesis

**Figure 3:**
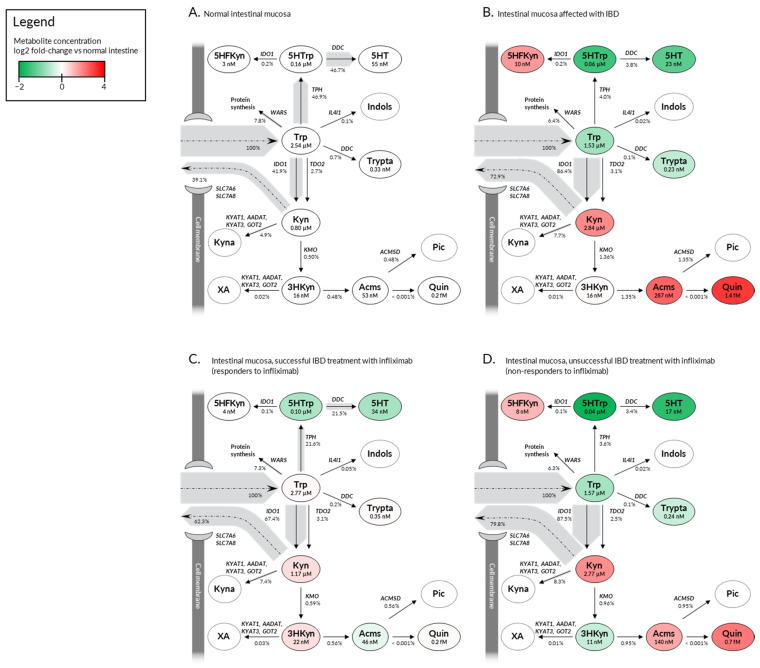
Predicted flux distribution for tryptophan metabolism in the intestine mucosa. (**A**) normal intestinal mucosa (healthy control); (**B**) intestinal mucosa affected with IBD; untreated or placebo treated patients; (**C**) intestinal mucosa successfully treated with infliximab; patients responding to the biologic therapy; (**D**) intestinal mucosa unsuccessfully treated with infliximab; patients non-responsive to the biologic therapy. For simplicity, only the main metabolic compounds and reactions are shown. The width of the shaded area around the arrow represents the predicted flux relative to tryptophan inflow. Relative fluxes and metabolite concentrations are indicated. Trp, tryptophan; Kyn, kynurenine; Kyna, kynurenic acid; 3HKyn, 3-hydroxy-kynurenine; XA, xanthurenic acid; Acms, 2-amino-3-carboxymuconate semialdehyde; Pic, picolinic acid; Quin, quinolinic acid; 5HTrp, 5-hydroxytryptophan; Trypta, tryptamine; 5HFKyn, 5-hydroxy-N-formylkynurenine; *ACMSD*, 2-amino-3-carboxymuconate semialdehyde-decarboxylase; *IL4I1*, interleukin 4 induced 1; *WARS*, tryptophanyl-aminoacyl-tRNA synthetase; *IDO1*; indoleamine 2,3-dioxygenase 1; *TOD2*, tryptophan 2,3-dioxygenase; *DDC*, aromatic l-amino acid decarboxylase; *TPH*, tryptophan hydroxylase; *KYAT1*, kynurenine aminotransferase 1; *AADAT*, aminoadipate aminotransferase; *KYAT3*, kynurenine aminotransferase 3; *GOT2*, glutamic-oxaloacetic transaminase 2; *KMO*, kynurenine 3-monooxygenase; *SLC7A5*, large neutral amino acids transporter small subunit 1; *SLC7A8*, large neutral amino acids transporter small subunit 2.

**Figure 4:**
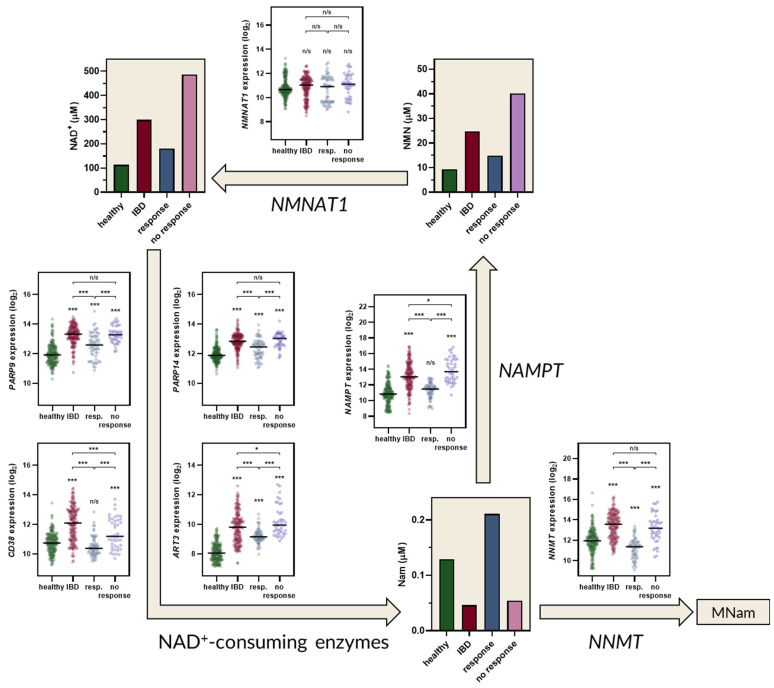
Predicted concentrations of NAD^+^ metabolites in the intestine mucosa of healthy controls, patients with inflammatory bowel disease (IBD), IBD patients responding to infliximab treatment (resp.), and IBD patients non-responding to infliximab treatment (no response). For simplicity, only the key elements of the NAD^+^ biosynthesis pathway are shown. Expression values of key enzymes used for pathway modeling are shown as scatter plots. Statistical analysis: one-way ANOVA with Tuckey’s post-hoc test. *, *p*-value < 0.05; ***, *p*- value < 0.001. Metabolites: Nam, nicotinamide; NAD, nicotinamide adenine dinucleotide; NMN, Nam mononucleotide; MNam, methyl-Nam. Enzyme-coding genes: *NMNAT1*, nicotinamide nucle-otide adenylyltransferase 1; *NAMPT*, nicotinamide phosphoribosyltransferase; *NNMT*, nico-tinamide N-methyltransferase; *PARP9/14*, poly(ADP-ribose) polymerase 9/14; *CD38*, cyclic ADP-ribose hydrolase; *ART3*, ecto-ADP-ribosyltransferase 3.

Trp metabolism is driven by an intricate network of reactions (Figure 2). The complexity originates from the fact that the same enzymes in the pathway metabolize multiple substrates, e.g., *KYNU*-encoded kynureninase catalases cleavage of Kyn into anthranilic acid (AA), but it can also process formyl-kynurenine (FKyn) and 3-hydroxy-kynurenine (3HKyn), although with varying enzyme activity. Moreover, some metabolites are processed by several different enzymes or transported using the same membrane carriers, e.g., both Kyn and Trp are substrates for SLC7A5 and SLC7A8 large amino acid transporters. Thus, alterations in gene expressions do not readily indicate pathological shifts of Trp metabolites’ concentrations. Therefore, a comprehensive mathematical model was needed to facilitate the identification of potential pathological changes in Trp metabolism during the development and treatment of IBD. Here, we utilized previously validated kinetic models that were adjustable using tissue-specific gene expression data. The models compassed kynurenine and 5HT pathways (Figure 3) and NAD^+^ metabolism (Figure 4). The models were fed with gene expression data from (I) normal human intestinal mucosa, (II) mucosa from non-treated patients affected with IBD, (III) mucosa from IBD patients successfully treated with infliximab (i.e., responders), and (IV) mucosa from IBD patients not responding to infliximab (Supplementary Figure S2, Figure 4). This allowed us to predict steady state concentrations and/or production rates (fluxes) of Trp and its key metabolites in the intestinal mucosa of IBD patients differentially responding to the treatment (Figure 3 and Figure 4).

Metabolite flux analysis indicated that, under normal conditions, mucosal Trp was almost equipotently processed by IDO1 (41.9%) and TPH (46.9%), with other enzymes being less involved (Figure 3A). This balance was disturbed in IBD, where Trp shuttled almost exclusively towards kynurenine pathway (89.5%), leading to a decrease in 5HT production and increase in Kyn levels (Figure 3B). Elevated Kyn fueled the formation of downstream metabolites, including Kyna, 2-amino-3-carboxymuconate semialdehyde (Acms), and quinolinate (Quin). IBD-related changes in kynurenine’s levels were largely reversed in the intestinal mucosa of infliximab responders (Figure 3C), but not in non-responders (Figure 3D). Interestingly, already low 5HT levels were predicted to be further suppressed in infliximab-treated patients who were resistant to the drug (Figure 3D).

To validate the simulations, the calculated metabolic changes were compared with concentrations of Trp metabolites measured in IBD patients and normal controls (Table 1). The employed models assume that Trp and Kyn are readily transported to and from tissues by membrane transporters SLC7A8 and SLC7A5. Thus, Trp and Kyn produced endogenously in organs should contribute to the plasma pool of these compounds. The intestine, the brain, and the liver are the key tissues responsible for Trp processing [15,16,17]. Consequently, the calculated levels of Trp and Kyn for these three organs were combined and compared with measured concentrations from the literature (Table 1). The calculated concentrations were within the ranges reported by Dudzińska et al. [18]. This indicates that the model correctly predicted how IBD-associated alterations in gene expression affect metabolite levels.

In parallel to tryptophan pathway analysis, concentrations of metabolites involved in NAD^+^ biosynthesis and turnover were predicted (Figure 4). Our calculations indicated that the level of NAD^+^ is increased in IBD patients by a factor of circa three. A similar increase in NAD^+^ concentration was observed in the dextran sulfate sodium (DSS) model of colitis in mice [19]. The calculated NAD^+^ concentration for responders and non-responders indicated that only successful treatment with infliximab leads to a decrease in NAD^+^ (Figure 4). Disease- and treatment-related changes in NAD^+^ levels were independent of nicotinamide nucleotide adenylyltransferase 1 (NMNAT1) activity, as its expression was not altered in IBD nor was it affected by infliximab (Figure 4). Instead, fluctuations in NAD^+^ levels followed the changes in the expression of nicotinamide phosphoribosyltransferase (NAMPT) and NNMT.

## 3. Discussion

Our work stems from the finding that *IDO1*, *KYNU*, and *HCAR3* are selectively upregulated in IBD and downregulated in response to successful infliximab treatment. The genes encode either enzymes involved in generation of kynurenines (*IDO1* and *KYNU*) or receptors for kynurenines (*HCAR3*). This encouraged us to adapt available models of Trp metabolism to predict the performance of the Trp metabolic pathway in the course and treatment of IBD. Our findings suggest that intestinal Trp metabolism in IBD shifts towards the kynurenine pathway, leading to the increased formation of Kyn and other kynurenines. The pathophysiological role of such metabolic imbalance is disputed in the literature. Two opposing concepts explaining the role of disturbed kynurenine pathway prevail: (a) enhancement of Trp processing via the kynurenine pathway constitutes a pathological condition that drives the disease’s progression, (b) kynurenine pathway activation is a part of the physiological compensatory mechanism intended to counterbalance the disease’s symptoms [20]. Our simulations demonstrated normalization of the kynurenine pathway specifically in infliximab responders. Unfortunately, this observation fits both concepts and will not help to decipher the role of kynurenine pathway imbalance in IBD. One may propose that Kyn metabolism returns to baseline in response to infliximab because the drug suppressed the pathological pathway; however, it is equally plausible that the compensatory action of the kynurenine pathway is no longer needed and undergoes physiological attenuation, since infliximab has already stopped the progression of the disease.

Despite difficulties in drawing ultimate conclusions, our work is the first to predict changes in Trp metabolites’ levels in response to IBD treatment. The concentration values obtained seem to match those described in the literature. Dudzińska et al. reported that experimentally quantified serum Trp levels dropped from 48.1 µM in controls to 35.6 and 37.8 µM in UC and CD patients, respectively [18]. Our calculations indicated that serum Trp concentration in healthy subjects is equal to approximately 46.4 µM and decreases to about 39.3 µM in IBD patients. This indicates that the applied models captured not only the qualitative change in Trp levels (decrease in serum), but correctly estimated the quantitative nature of that change. Similar observations were made regarding Kyn. Several reports indicated an increase in plasma Kyn levels or in Kyn/Trp ratio in IBD patients vs. normal controls [18,21,22]. Our modeling recapitulated that effect. 

To further corroborate the model, literature databases were searched for reports on Trp and Kyn levels in IBD patients treated with infliximab. A single study demonstrating Trp levels in response to infliximab was identified [21]. The authors noticed that successful therapy with infliximab led to a significant and sustained increase in serum Trp levels in comparison to Trp levels measured before the beginning of the treatment [21]. However, there was no Trp reconstitution in non-responders [21]. Our calculations captured the same treatment-associated dynamics of Trp (Figure 3). In responders, mucosal Trp levels increased from 1.53 µM at baseline to 2.77 µM, which roughly should generate an increase of around 9 µM of Trp in serum. This represents a median increase of around seven micromolar units (25th percentile: ~3 µM; 75th percentile: ~17 µM), as measured in the serum of infliximab responders six months after successful therapy [21]. According to our calculations, non-responders should generate little to no change in Trp levels (both in the intestinal mucosa and serum) compared to baseline (Figure 3). This is similar to the results of Nikolaus and colleagues, who reported that serum Trp levels return to baseline after a short initial increase observed in non-responders [21]. Taken together, this suggest that the model correctly deciphers metabolite levels in differentially responding patients.

IBD-related changes in the 5HT branch of Trp metabolism were reported in the literature, however, there was no consistency in the findings [23]. Some studies reported a drop [24,25,26] whereas others noted a rise [27,28] in 5HT content in affected individuals compared to healthy controls. Our modeling predicted a suppression of 5HT and its precursor 5-hydroxy-tryptophan (5HTrp) in IBD patients that further increases in the individuals unsuccessfully treated with infliximab. However, it needs to be noted that the employed model did not take into account the transport of 5HT though the cell membrane, which may affect the steady state concentration of this metabolite. This is a clear limitation of the study, as transport of Trp metabolites is one of the factors shaping their intracellular and serum concentrations [29,30]. Unfortunately, substrate kinetics for SLC6A4, a 5HT transporter, are unavailable (UniProt: P31645). Similarly, SLC22A6 and SLC22A8 organic anion transporters were implicated in the uptake of Kyna in the *Xenopus laevis* oocyte expression system [31]. Yet again, lack of kinetic data withheld us from incorporating the transmembrane transport of Kyna into the model. Another limiting aspect of the pathway reconstruction carried out here was the lack of calculations regarding the effect of IL4I1-dependent formation of Kyna, due to a lack of kinetic data. IL4I1 was only recently discovered to contribute to Kyna production [14], which might be of special importance here, as *IL4I1* expression correlates with the expression of *IDO1* in IBD patients.

Kyna and other kynurenines produced along the kynurenine pathway are biologically active molecules that exert their function through interaction with receptors. Thus, tracking the changes in the production of kynurenines and analyzing the expression pattern of their receptors may shed some light on the function of the kynurenine pathway in IBD. According to our predictions, Kyn concentration in the intestinal mucosa rises three-fold in IBD. Kyn is recognized as an agonist of the aryl hydrocarbon receptor (AHR). AHR is known as a ligand-activated transcription factor that integrates environmental, dietary, intestinal, microbial, and endogenous metabolic signals to control complex transcriptional programs in ligand-specific, cell type-specific, and context-specific manners. Its activity affects several physiological and pathological processes, including carcinogenesis [32]. It is widely accepted that AHR is involved in IBD, and this idea is consistently presented in topical reviews on the subject [33,34,35,36,37]. In the intestine, an anti-inflammatory and regenerative outcome of AHR activation was reported. Nguyen and colleagues showed that AHR negatively regulates dendritic cells’ immunogenicity via a kynurenine-dependent mechanism, leading to the induction of naïve T-cell differentiation into Treg and Th17 cells in vitro [38]. Similarly, Abron et al. showed that AHR activation induces regulatory T cells and ameliorates experimental colitis in mice [39]. Gasaly and co-workers considered that stimulation of mucosal AHR enhances intestinal epithelial barrier function [40]. Wang et al. postulated that activation of AHR regulates intestinal damage and inflammation to maintain intestinal integrity and homeostasis [41]. These findings support the notion of the compensatory role of Kyn in IBD.

Apart from Kyn, AHR can be activated by Kyna [42]. Kyna is formed by kynurenine aminotransferases (KATs) encoded by *KYAT1*, *AADAT*, *KYAT3*, and *GOT2* genes. Our simulation showed that the production rate of Kyna (KAT flux) increases from 4.9% in healthy controls to 7.7% in IBD patients. Unfortunately, the applied model is unable to calculate steady state concentrations for Kyna, as it is an external metabolite, due to a lack of information concerning the transport and secretion of Kyna. Nevertheless, increased flux suggests increased availability of Kyna for intracellular and/or paracrine signaling. This is especially important, as Kyna was demonstrated to act as a regulator in inflammation. In particular, Kyna suppressed anti-inflammatory IL10 and contributed to colitis in mice [43], but attenuated the TNF-α expression in human mononuclear cells activated by heat-inactivated *Staphylococcus aureus* [44]. Interestingly, Kyna activity may originate from binding to receptors other than AHR. In fact, Kyna was demonstrated to activate G protein-coupled receptor 35 (GPR35) [45] and HCAR3 [10]. Both GPR35 and HCAR3 couple with G proteins, but HCAR3 is exclusive to higher primates [46]. The fact that rodents lack one of the receptors for Kyna may undermine the translatability of murine IBD models. HCAR3 might be of particular importance, as its expression closely follows the expression of IDO1 in the intestinal mucosa of IBD patients. According to the Human Protein Atlas, HCAR3 is abundantly expressed in neutrophils, monocytes, and basophils. Apart from blood cells, it can be found in bone marrow and the spleen. Based purely on the expression profile, one could expect HCAR3 to have immunomodulatory properties. It should be noted that hyporesponsiveness of the immune system and an anti-inflammatory effects were suggested upon activation of HCAR3 [47,48]. It was also indicated that HCAR3 could be involved in sepsis [49] and coronary heart disease [50]. A single study identified HCAR3 as a potential target in colorectal cancer [51]. Experimental evidence is, however, scarce [46]. Assuming functional similarity of HCAR2 and HCAR3, the anti-inflammatory function of HCAR3 is expected [52]. Moreover, HCAR3 couples to inhibitory Gα_i_ to decrease cAMP levels, and the cAMP signaling is known to regulate innate and adaptive immune cell activities [53]. At present, the role of HCAR3 and its involvement in IBD pathophysiology is completely unknown. Thus, our finding indicates the need for an urgent investigation of this issue. According to the Guide to Pharmacology Database, no antagonists of HCAR3 are known; however, multiple chemical species of agonists were described [54]. The lack of pharmacological blockers of HCAR3 may hamper the research by limiting the available options to suppress the activity of the receptor to genetic inhibition, e.g., by the means of RNAi.

Our calculations demonstrated that an increase in Kyn is not accompanied by elevation of 3HKyn. This can be explained by the increased production of its downstream metabolites, Acms and Quin. Quin serves as a precursor of NAD^+^, possibly contributing to enhance de novo NAD^+^ production. It should be noted, however, that the salvage synthesis pathway is usually preferred and prevails over de novo production from Trp. As revealed by modeling, the salvage pathway for NAD^+^ production is clearly activated in IBD. Predicted mucosal NAD^+^ concentration rises from 113 µM in healthy controls to 299 µM in IBD patients. The enhancement of *IDO1* expression is accompanied by an increase in the expression of NAMPT and NAD-consuming enzymes: PARP9, CD38, PARP14, and ART3. Interestingly, enzymes that catabolize NAD^+^ have been implicated in pro-inflammatory processes. For instance, CD38 contributes to pro-inflammatory phenotypes in innate immune cells [55], and PARP9 initiates and amplifies type I interferon production in response to RNA viruses [56]. Thus, an NAD^+^ surplus in IBD may contribute to a rise in intestinal inflammation through the increased activity of NAD-consuming enzymes.

NAMPT catalyzes the rate-limiting step of the NAD^+^ salvage pathway, maintaining NAD^+^ supply. NAMPT is strongly upregulated in patients with IBD. It has been demonstrated in mice that the blocking of NAMPT enzymatic activity with the small-molecule inhibitor FK866 ameliorates experimental colitis [19]. NAMPT inhibition coincided with NAD^+^ depletion, the inhibition of NAD^+^ consuming enzymes, and suppressed cytokine release, indicating that targeting the NAD^+^ salvage pathway constitutes a promising target for IBD management [19]. 

An increase in NAD^+^ resulting from NAMPT upregulation is expected, as this enzyme catalyzes the transformation of nicotinamide (Nam) into nicotinamide mononucleotide (NMN), precursor of NAD^+^. However, the fact that modulation of NAD^+^ concentration coincided with synchronous changes in NNMT might be counterintuitive, as this enzyme methylates Nam and prompts its excretion from the body, consequently diminishing the pool of precursors for NAD^+^ synthesis. Surprisingly, NNMT may display entirely different functionality than to just remove Nam from NAD^+^ metabolism [13]. Most NAD^+^-consuming enzymes are inhibited by their product, Nam. As NNMT directs the removal of excess Nam, it enables higher NAD^+^ consumption [13]. At the same time, a high substrate affinity of NAMPT [13], combined with its upregulation in IBD, ensures the maintenance of a high NAD^+^ concentration. Taking into account the fact that NNMT inhibition may have a similar suppressive effect on NAD^+^ production as an NAMPT blockade, it might be interesting to consider NNMT as another drug target in IBD. Exploitation of NNMT is especially appealing, as aberrant NNMT expression has been linked with cancer, metabolic diseases, neurodegeneration, and inflammatory conditions [57]. Importantly, multiple classes of NNMT inhibitors were synthesized and are available for testing in IBD models [57].

## 4. Materials and Methods

### 4.1. Software

Transcriptome analyses were performed using the Genevestigator v7.4.1 (Nebion AG, Zurich, Switzerland) tool package [58]. The modeling of Trp metabolism was performed using the COPASI 4.33 pathway simulator developed at University of Virginia, University of Heidelberg, and University of Connecticut School of Medicine [59].

### 4.2. Identification of Genes Regulated during IBD Development and Treatment

The Perturbations tool from the Genevestigator Gene Search toolset was used to identify genes that are specifically up- or down-regulated in a chosen set of conditions and which minimally change in all other conditions. The analysis was conducted on data from Affymetrix Human Genome U133 Plus 2.0 Array and limited to intestinal samples. The Gene Expression Omnibus (GEO) dataset on mucosal expression profiling in patients with IBD before and after treatment with infliximab (GEO:GSE16879) was used for gene identification [60], while the remaining datasets were used as background. The minimum target |log_2_ ratio| was 1.6, which allowed us to select the 20 most affected genes.

### 4.3. Correlation Analysis

Data on a relative change (log_2_-ratio) in the expression of Trp-related genes in the context of IBD were extracted from the Genevestigator database using the Perturbations tool. The relative gene expression values originated from microarray platforms (*n* = 204) and RNA sequencing experiments (*n* = 153). The log_2_-ratios for the *IDO1* gene from all relevant experiments were plotted against log_2_-ratios for all other Trp-related genes from the same experiments. Linear regression was conducted to fit a straight line to the data and to calculate a slope value. In addition, the squared Pearson correlation coefficient (R^2^) was computed. Correlation was considered strong for R^2^ ≥ 0.6. Moderate correlation occurred when R^2^ < 0.6 and R^2^ ≥ 0.4.

To demonstrate whether the correlations observed for IBD were specific to the disease, similar analysis was performed using data from 177 gene expression studies on human intestinal neoplasms.

### 4.4. Tryptophan Metabolism Modeling

The concentrations and fluxes of key Trp metabolites were estimated using two previously described models: (I) the model of Trp metabolism encompassing Kyn and 5HT branches [12], and (II) the NAD^+^ biosynthesis model [13]. The *sbml* files of the models were downloaded from the BioModels database [61]. The files were available under the following identifiers: MODEL1310160000 and MODEL1905220001. Both models could integrate already provided kinetic data for relevant enzymes with tissue-specific expression levels. Model adjustment with gene expression values and all calculations were performed using COPASI application.

Relevant microarray gene expression values were extracted using Genevestigator. Intestinal gene expression levels originated from four groups of subjects: normal controls (*n* = 186), patients diagnosed with IBD that were either treatment-naïve or exposed to a placebo (*n* = 162), IBD patients successfully treated with infliximab (*n* = 60), and IBD patients producing no response to infliximab treatment (*n* = 42).

The model of Trp metabolism (kynurenine and 5HT pathways) received no modifications apart from adjustment for tissue- and treatment-specific gene expression. The model of NAD^+^ was modified by changing the scaling factor to 10^−5^ in order to obtain NAD^+^ concentrations within the high micromolar range observed in mammalian cells [62,63,64]. The original version of the NAD^+^ metabolism model contained single prototypical NAD^+^-consuming enzyme (SIRT) at expression scaled to 1:40 of NAMPT level. Here, we averaged the expression values of all genes coding for NAD^+^-consuming enzymes (*SIRT1*, *SIRT2*, *SIRT3*, *SIRT4*, *SIRT5*, *SIRT7*, *PARP1*, *PARP2*, *CD38*, *BST1*, *ART1*, *ART3*, *ART4*, *ART5*, *PARP3*, *PARP4*, *PARP6*, *TIPARP*, *PARP8*, *PARP10*, *PARP11*, *PARP12*, *PARP14*, *PARP15*, *PARP9*, *PARP16*, *TNKS*, *TNKS2*, *TRPT1*) and kept expression value at the same rate (1:40) in relation to NAMPT in the healthy control dataset. In the three remaining datasets (IBD, IBD responders, and IBD non-responders) we multiplied the average expression value for NAD^+^-consuming enzymes by a scaling factor equal to:

(1) $$\frac{E_{NAMPT\left(control\right)}}{40}\times \frac{1}{\bar{E_{NAD^{+}-consuming\;enzymes\;\left(control\right)}}},$$

where *E* refers to expression level for a given gene in the control group.

### 4.5. Statistical Analysis

Differences in mean expression values were analyzed using one-way analysis of variance (ANOVA) followed by a Tukey’s post-hoc test. Prism 8.4.3 (GraphPad Software Inc., San Diego, CA, USA) was used to perform calculations and to draw scatter plots. *p*-values below 0.05 were marked with a single asterisk (*) and were considered significant. *p*-values < 0.01 were marked with a double asterisk (**), and *p*-values < 0.001 were marked with a triple asterisk (***).

## 5. Conclusions

The reprogramming of Trp and NAD^+^ metabolism has been observed in numerous conditions, ranging from cancer to inflammatory disorders [21,65]. Consequently, interest in therapeutically targeting the kynurenine pathway and NAD^+^ biosynthesis has been triggered and has generated several promising drug candidates [19,66,67]. This study shows that altered Trp metabolism normalize in patients successfully treated with infliximab and suggests HCAR3 and NNMT as potential IBD drug targets.

## Figures and Tables

**Table 1:** Comparison between calculated and experimentally measured concentrations of tryptophan (Trp) and kynurenine (Kyn) in human serum.

		Calculated Intestine Level ^1^	Calculated Brain Level [12]	Calculated Liver Level [12]	Calculated Serum Level ^2^	Measured Serum Level [18]
Trp	normal	2.5 µM	3.9 µM	0.1 µM	46.4 µM	48.1 ± 13.1 µM
	IBD	1.5 µM			39.3 µM	35.6 ± 11.1 µM (UC) 37.8 ± 9.4 µM (CD)
Kyn	normal	0.8 µM	0.5 µM	2.6 µM	3.9 µM	2.9 ± 1.2 µM
	IBD	2.8 µM			5.9 µM	5.5 ± 5.2 µM (UC) 8.4 ± 7.4 µM (CD)

^1^ As presented on Figure 3A,B. ^2^ Trp and Kyn are readily transported to and from the blood by membrane transporters. Thus, serum levels were calculated as a sum of concentrations’ values modeled for the intestine (Figure 2), the brain [12], and the liver [12]. For Trp, it was taken into account that the concentrations of free Trp available to the cells correspond to about 14% of the total serum Trp [12].

## Data Availability

Raw data analyzed in this study are available through Genevestigator software that can be obtained at genevestigator.com (accessed on 4 November 2021).

## Supplementary Materials

All data are available online at https://www.mdpi.com/article/10.3390/ijms222413497/s1.
