# Supplementary material for: Alterations in Kynurenine and NAD+ Salvage Pathways during the Successful Treatment of Inflammatory Bowel Disease Suggest HCAR3 and NNMT as Potential Drug Targets
Source: Int J Mol Sci. 2021 Dec 16;22(24):13497. doi: 10.3390/ijms222413497 (PMC8705244; doi:10.3390/ijms222413497)
Supplement: Supplementary file 1 [file ijms-22-13497-s001.zip › ijms-1473713-supplementary.pdf]

# Alterations in Kynurenine and NAD+ Salvage Pathways During the Successful Treatment of Inflammatory Bowel Disease Suggest HCAR3 and NNMT as Potential Drug Targets

Wnorowski et al. 2021

Supplementary Materials

Figure S1: Correlation between changes in *IDO1* expression vs. change in the expression of genes related to tryptophan metabolism in inflammatory bowel disease (IBD) or intestinal neoplasms.

*page 2*

Figure S2: Expression profiles of genes related to tryptophan metabolism and $NAD^+$ biosynthesis in the intestinal mucosa of healthy controls, patients diagnosed with inflammatory bowel disease (IBD), patients responding to infliximab treatment, and non-responders to infliximab.

*page 3*

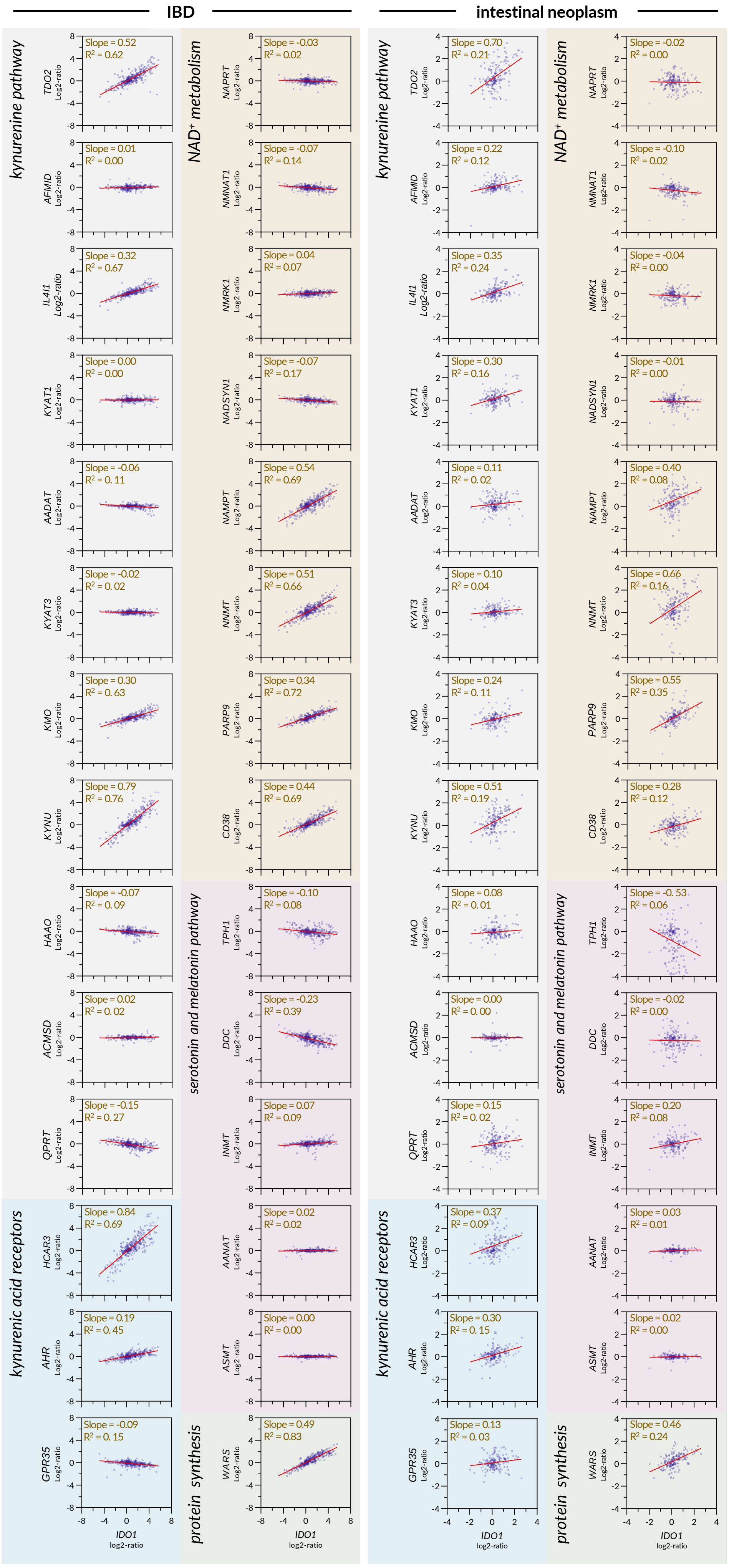


Figure S1. Correlation between changes in *IDO1* expression vs. change in the expression of genes related to tryptophan metabolism in inflammatory bowel disease (IBD) or intestinal neoplasms.

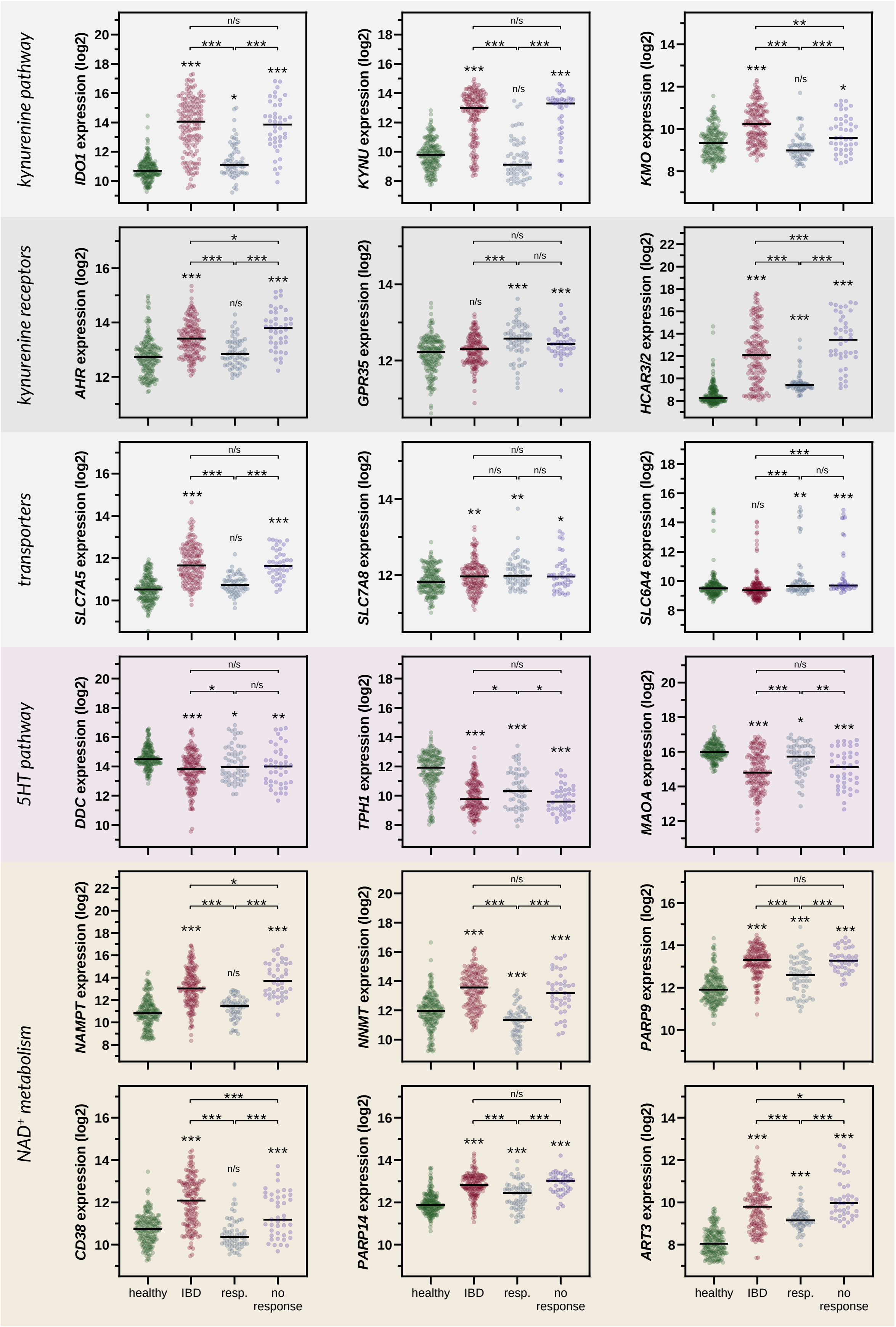


Figure S2. Expression profiles of genes related to tryptophan metabolism and NAD⁺ biosynthesis in the intestinal mucosa of healthy controls (*healthy*), patients diagnosed with inflammatory bowel disease (*IBD*), patients responding to infliximab treatment (*resp.*), and non-responders to infliximab (*no response*).
